# Origin, Diversity, and Evolution of Telomere Sequences in Plants

**DOI:** 10.3389/fpls.2020.00117

**Published:** 2020-02-21

**Authors:** Vratislav Peska, Sònia Garcia

**Affiliations:** ^1^ Department of Cell Biology and Radiobiology, The Czech Academy of Sciences, Institute of Biophysics, Brno, Czechia; ^2^ Institut Botànic de Barcelona (IBB, CSIC-Ajuntament de Barcelona), Barcelona, Spain

**Keywords:** *Allium*, *Cestrum*, circular chromosomes, *Genlisea*, green algae, linear chromosomes, telomerase, telomeres

## Abstract

Telomeres are basic structures of eukaryote genomes. They distinguish natural chromosome ends from double-stranded breaks in DNA and protect chromosome ends from degradation or end-to-end fusion with other chromosomes. Telomere sequences are usually tandemly arranged minisatellites, typically following the formula (T_x_A_y_G_z_)_n_. Although they are well conserved across large groups of organisms, recent findings in plants imply that their diversity has been underestimated. Changes in telomeres are of enormous evolutionary importance as they can affect whole-genome stability. Even a small change in the telomere motif of each repeat unit represents an important interference in the system of sequence-specific telomere binding proteins. Here, we provide an overview of telomere sequences, considering the latest phylogenomic evolutionary framework of plants in the broad sense (Archaeplastida), in which new telomeric sequences have recently been found in diverse and economically important families such as Solanaceae and Amaryllidaceae. In the family Lentibulariaceae and in many groups of green algae, deviations from the typical plant telomeric sequence have also been detected recently. Ancestry and possible homoplasy in telomeric motifs, as well as extant gaps in knowledge are discussed. With the increasing availability of genomic approaches, it is likely that more telomeric diversity will be uncovered in the future. We also discuss basic methods used for telomere identification and we explain the implications of the recent discovery of plant telomerase RNA on further research about the role of telomerase in eukaryogenesis or on the molecular causes and consequences of telomere variability.

## Introduction

Telomeres are nucleoprotein structures at the very ends of linear eukaryotic chromosomes. They solve two major end-problems at the same time. The first is about chromosome end protection. It is estimated that normal human cells must repair at least 50 endogenous double-stranded breaks (DSBs) per cell per cell-cycle (Vilenchik and Knudson, 2003). Telomeres distinguish the natural chromosomal ends from harmful DSBs and prevent their ectopic repair, e.g., by end-to-end fusions of chromosomes (vanSteensel and deLange, 1997). The second is the end-replication problem that deals with the maintenance of proper telomere lengths. This was recognized independently by two researchers (Watson, 1972; Olovnikov, 1973). Since replicative DNA-dependent DNA polymerases cannot complete DNA synthesis at the very ends of chromosomes, compensation for replicative telomere sequence loss must come from an RNA-dependent DNA polymerase. This enzyme, called telomerase, together with the first telomere minisatellite sequence, was discovered in the ciliate *Tetrahymena* (Blackburn and Gall, 1978; Greider and Blackburn, 1985). However, this is only one aspect of telomere length maintenance. The epigenetic regulation of telomere length homeostasis, including interaction of long noncoding telomeric repeat containing RNA and exonuclease activity pathways, have also been extensively studied due to its therapeutical potential (Wellinger et al., 1996; Polotnianka et al., 1998; Pfeiffer and Lingner, 2012).

**Figure 1:**
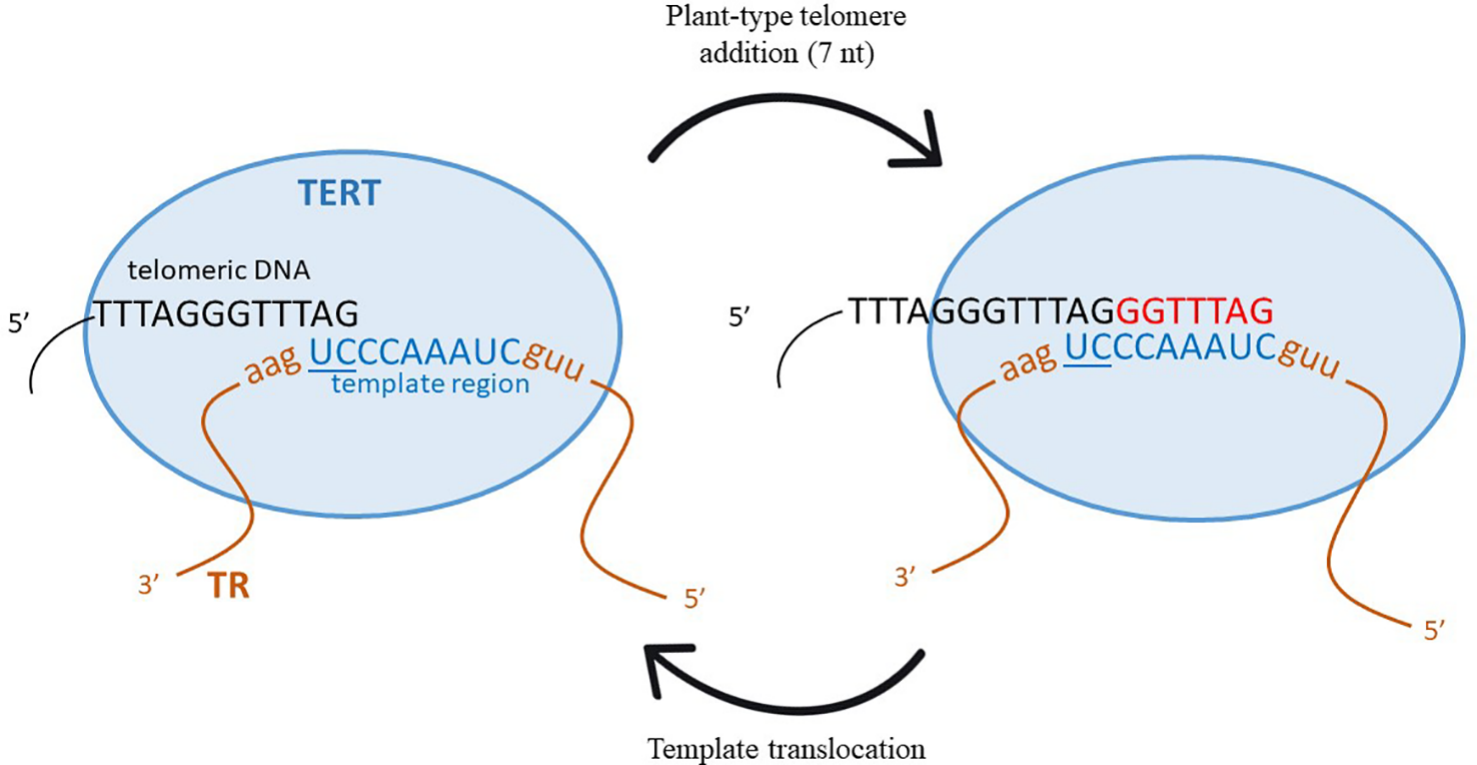
Schematic representation of the telomerase activity cycle with the *Arabidopsis*-type telomere template. TERT, Telomere Reverse Transcriptase; TR, telomerase RNA subunit. Figure based on Sekhri (2014).

Telomerase, the enzyme in charge of adding telomere repeat sequences to the 3' end of telomeres, is a conserved complex enzyme with numerous components [its structure has been recently reviewed by (Wang et al., 2019), and specifically for plants, by (Majerska et al., 2017)]. In principle, only two main components are essential for telomerase enzymatic activity, a catalytically active protein component, called telomere reverse transcriptase (TERT), and a template component, formed by the telomerase RNA subunit (TR). While TERT is evolutionarily quite well conserved, TR is very variable, with lengths ranging from ca. 150 nt (*Tetrahymena*) to more than 2,000 nt (fungi from genus *Neurospora*). Only a short region in the whole TR molecule serves as a template for newly synthesized telomere DNA (Greider and Blackburn, 1985; Qi et al., 2013). This region in TR is usually formed by a complete telomere motif followed by a partial one, the latter serving as an annealing region for the existing telomere DNA. Although, in principle, only a single extra nucleotide is needed (as a partial motif), usually more than one is found. For example, two extra nucleotides form the annealing motif in mice or five in human (Blasco et al., 1995; Feng et al., 1995). In plants, however, the size of the template region is variable, e.g., two in *Arabidopsis thaliana*, seven in *Arabis* sp. or six in *Nicotiana* (Fajkus et al., 2019). The other TR regions have structural, regulatory and protein interactive functions [reviewed in (Podlevsky and Chen, 2016)]. See also a schematic depiction of telomerase and its activity cycle in **Figure 1**.

## How Variable Are Telomere Sequences?

Telomere sequences are usually short minisatellites tandemly arranged, typically following the formula (T_x_A_y_G_z_)_n_. The minisatellite arrangement originates from the way in which telomerase synthesizes the DNA, in short, and mostly identical motifs, one by one. Several hypotheses consider that such an arrangement is important because it promotes the recognition of telomere specific proteins by homo- and heterodimers [e.g., (Hofr et al., 2009; Visacka et al., 2012)] and for the potential to form G-quadruplexes that may stabilize chromosome ends or serve as substrates for telomere-specific proteins (Spiegel et al., 2020; Tran et al., 2013). Telomere sequences are well conserved through evolution, and large groups of organisms use the group-typical telomere motif to build their telomere DNA. A gradually increasing number of studies and large screenings have shown that all tested vertebrates and many basal metazoans use TTAGGG (Meyne et al., 1989; Traut et al., 2007) while Euarthropoda (arthropods), including Hexapoda (insects), have TTAGG (Frydrychova et al., 2004; Vitkova et al., 2005). Steadily, numerous exceptions are accumulating over time, e.g., (A(G)_1-8_) in *Dictyostelium* (Emery and Weiner, 1981), TTAGGC in *Ascaris lumbricoides* (Nematoda) (Muller et al., 1991), TCAGG in Coleoptera (beetles) (Mravinac et al., 2011), TAGGG/TAAGG/TAAGGG in *Giardia* (diplomonads) (Uzlikova et al., 2017), or TTNNNNAGGG in *Yarrowia* clade (yeasts) (Cervenak et al., 2019). Moreover, telomerase-independent systems, in which the minisatellite telomere sequence has been lost and substituted by complex repeats, are represented, for example, by *Diptera* and Chironomidae (reviewed in (Mason et al., 2016)). For a general review on eukaryotic telomere sequence see (Fajkus et al., 2005; Fulneckova et al., 2013).

Telomere composition in plants is even more diverse. Here we use the term “plants” in a broad sense, also known as Archaeplastida or kingdom Plantae *sensu lato*, and comprising Rhodophyta (red algae), Glaucophyta, the Chlorophyte algae grade and the Streptophyte algae grade (altogether known as green algae), and Embryophyta (land plants) (One Thousand Plant Transcriptomes Initiative, 2019). The typical telomere plant sequence is TTTAGGG, also called *Arabidopsis*-type (or simply, plant-type) since it was discovered in *Arabidopsis thaliana* (Richards and Ausubel, 1988) and now in many other species across almost all plant orders. Although TTTAGGG is still the most frequent, there is significant variability in telomere sequences in red and green algal lineages. As for red algae (Rhodophyta), telomere sequence information is mostly missing or fragmentary, although some telomere candidates have been discovered *in silico*, such as AATGGGGGG for *Cyanidioschyzon merolae* (Nozaki et al., 2007), TTATT(T)AGGG for *Galdieria sulphuraria* (Fulneckova et al., 2013); TTAGGG has been found in genomic reads of *Porphyra umbilicalis* (Fulneckova et al., 2013), but more evidence is needed to confirm their terminal position on chromosomes. Telomere diversity in green algae reflects both dynamic changes and its paraphyletic character. Although TTTAGGG prevails in Chlorophyta, such as in genera *Ostreococcus* (Derelle et al., 2006) and *Chlorella* (Higashiyama et al., 1995), many other divergent motifs have been detected there too, such as TTAGGG in genus *Dunaliella* and *Stephanosphaeria* (Fulneckova et al., 2012), and TTTTAGGG in *Chlamydomonas* (Petracek et al., 1990). In basal Streptophyta (Klebsormidiophyceae) progressive changes in motifs from TTTAGGG to TTTTAGGG and TTTTAGG have been described. The presence of TTAGGG in Rhodophyta and Glaucophyta leads to the hypothesis that this is the ancestral motif in plants (Archaeplastida) (Fulneckova et al., 2013).

Concerning land plants, one of the first screenings performed showed that the *Arabidopsis*-type sequence was the most common and was mostly conserved through their phylogeny (Cox et al., 1993; Fuchs et al., 1995), although some of these authors had already detected several exceptions in the family Amaryllidaceae (former Alliaceae), in which the *Arabidopsis*-type sequence was absent in several species. Later, the first telomere sequence unusual for land plants, the vertebrate-type TTAGGG, was characterized in *Aloe* and in some other Asparagales (Weiss and Scherthan, 2002; Puizina et al., 2003; Sykorova et al., 2003c). A hypothesis about repeated losses and recoveries of the TTTAGGG and TTAGGG telomere sequence in Asparagales was formulated (Adams et al., 2001). With the postrefinement of order Asparagales in the APGIII (Angiosperm Phylogeny Group 2009) (Bremer et al., 2009), it was shown that only two major evolutionary switches in telomere sequence composition occurred (rather than several repeated losses and gains), in the following order: the first one in family Iridaceae, in which a shift from the plant-type TTTAGGG to the vertebrate-type TTAGGG happened, followed by families Xeronemataceae, Asphodelaceae and the core Asparagales (including Amarillidaceae s.l and Asparagaceae s.l.); and the second one within subfamily Allioideae (formerly treated as a separate family, Alliaceae) in which a completely new telomere sequence emerged, CTCGGTTATGGG (Fajkus et al., 2016). Outside Asparagales, new telomere sequences have also been detected in land plant groups as disparate as (i) Solanaceae, in which the telomere sequence of *Cestrum elegans* TTTTTTAGGG was described (Sykorova et al., 2003a; Sykorova et al., 2003b; Peska et al., 2008; Peska et al., 2015) and (ii) Lentibulariaceae, where genus *Genlisea* showed a remarkable diversity with some species characterized by the *Arabidopsis*-type telomere repeats while others exhibited intermingled sequence variants TTCAGG and TTTCAGG (Tran et al., 2015).

**Figure 2:**
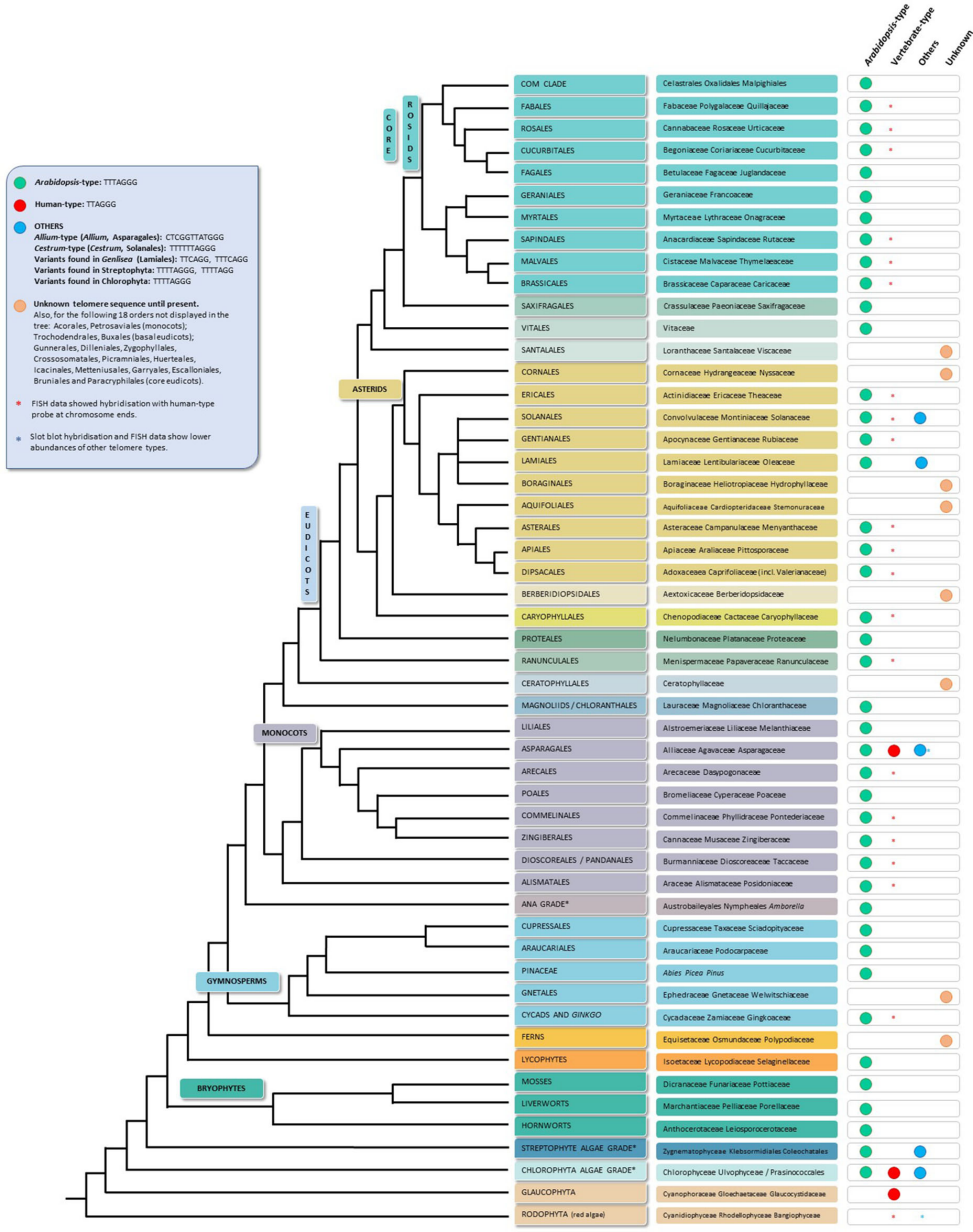
Telomere motifs in Archaeplastida (plants in the broad sense), based on the APG IV (The Angiosperm Phylogeny Group 2016) and on the One Thousand Plant Transcriptomes Initiative (2019). Branch lengths do not express real time scales. For simplicity and to save space, certain polyphyletic “groups” (grades) marked with an asterisk in the tree have been represented by a single branch; for the same reason, several minor orders (listed in the blue square at the left upper side of the figure) are not depicted on the tree. The first tip label usually refers to plant orders and in a few cases, to divisions, grades and even families; the second label displays representative families and in a few cases, representative orders or genera.

Despite all the telomere motif exceptions detected, the real diversity in telomeric sequences in land plants is probably greatly underestimated. A recent publication (Vitales et al., 2017), in which a screening of land plant telomere sequences was performed, found that telomere sequences were only known clearly for less than 10% of the species and 40% of the genera contained in the Plant rDNA database (www.plantrdnadatabase.com), a resource providing molecular cytogenetics information on land plants (Garcia et al., 2012). A summary of telomere sequence distribution in plants, following APG IV (The Angiosperm Phylogeny Group, 2016) (Byng et al., 2016), as well as the most recent plant phylogeny (One Thousand Plant Transcriptomes Initiative, 2019) is found in **Figure 2**.

## From Screenings to Discovery: How Telomeric Motifs Can Be Identified?

The evidence that a given candidate sequence is a real telomeric one includes several steps that properly declare its localization at all chromosomal termini, and eventually the involvement of telomerase in its synthesis. Molecular cytogenetics (mostly by Fluorescence *in situ* Hybridization, FISH) has become important for visualizing the terminal localization of labeled probes of candidate sequences at all chromosomal termini. However, standalone FISH it is not enough to prove the very terminal position. For example, AcepSAT356 [a 356bp-long satellite from *Allium cepa*, (Peska et al., 2019)] was proposed in onion as the telomere candidate, based on results from FISH analysis (Pich and Schubert, 1998). Nevertheless, its apparent terminal location by *in situ* has never been convincingly linked to telomere function. Actually, the discovery of the *Allium* minisatellite telomere sequence CTCGGTTATGGG and telomerase would mean that AcepSAT356 is subterminal (Fajkus et al., 2019). Positive FISH telomeric signals can also mask tiny changes in telomere motifs such as single nucleotide polymorphisms, or false-negative results may result from short telomeres being beneath the detection limit of the technique.

There are two additional approaches that determine the terminal position at greater resolution than FISH; these are based on exonuclease BAL31 activity. The first is the classical Terminal Restriction Fragment (TRF) analysis, in which samples treated by BAL31 show progressive shortening of terminal fragments and a decrease in signal intensity with increasing time of exonuclease treatment. The subsequent analysis of fragment lengths is performed by Southern-blot hybridization (Fojtova et al., 2015). The second is comparative genome skimming (NGS data) of nondigested and BAL31-digested genomic DNA, in parallel. In the BAL31 treated dataset, there is a significant under-representation of telomere sequences, therefore the terminal sequences are identified by comparison with the untreated dataset, using bioinformatics tools RepeatExplorer or Tandem Repeats Finder [a pipeline called BAL31-NGS (Benson, 1999; Novak et al., 2010; Peska et al., 2017)].

The other important test of a given telomere sequence candidate in a species is the demonstration of telomerase activity. In this, a useful experimental approach, developed first for human cells, is the Telomere Repeat Amplification Protocol (TRAP) (Kim et al., 1994), followed by sequencing of the detected products (Peska et al., 2015; Fajkus et al., 2016), which is a little less sensitive to false-positive results than FISH. All these methods, including FISH (Fuchs et al., 1995; Shibata and Hizume, 2011) and others such as slot-blot hybridization (Sykorova et al., 2003c), and TRAP (Fulneckova et al., 2012; Fulneckova et al., 2016), can be used to screen for telomeres across wide groups of complex organisms, including plants. However, only a combination of suitably chosen methods can convincingly lead to a conclusion about the telomere function of a candidate sequence, since results base on a single approach might be misleading. A more complete overview of the strategies for *de novo* telomere candidate sequence identification, including the very first attempt in *Tetrahymena* (Greider and Blackburn, 1985) are summarised in a methodological article, with emphasis on the NGS approach used in plants with extremely large genomes (Peska et al., 2017).

## Is There Homoplasy in Telomere Sequences?

The ancestral telomere sequence is thought to be TTAGGG and is the most commonly found across the tree of life (Fulneckova et al., 2013). Yet, it seems clear that the frequency of homoplasy in telomere motif evolution is relatively high. For example, short, simple motifs like the plant-type TTTAGGG have appeared independently and repeatedly in cryptomonads, oomycete fungi, and alveolates; similarly, the vertebrate-type TTAGGG has emerged secondarily in certain groups of plants (Asparagales, Rodophyta and Chlorophyta algae) (Sykorova et al., 2003c; Fulneckova et al., 2012; Fulneckova et al., 2013; Somanathan and Baysdorfer, 2018). The reason some telomere sequences have emerged more frequently than other, usually more complex sequences is probably related to selection pressures, which would favor accuracy for a particular sequence-specific DNA-protein interaction (Forstemann et al., 2003). If there was a change in each telomere motif, interference in the telomeric nucleoprotein structure would necessarily lead to genome instability. This is the reason telomere sequences are so evolutionary stable, comprising very few novel and successful sequences, a pattern consistent with the idea of repeated losses and the emergence of the typical telomere sequences, as proposed for Asparagales (Adams et al., 2001).

The finding of homoplasy across telomere sequences raises the question, what are the molecular causes and processes taking place during these shifts? A change in telomere sequence, despite seeming trivial in some cases (e.g., one extra T), may cause serious interference with genome integrity, because of a disturbed balance in the telomere DNA-protein interactions. It is also unclear whether a change in telomere sequence may have any evolutionary advantage; in this regard, (Tran et al., 2015) suggested that the appearance of a “methylatable” cytosine in a G-rich telomere strand would raise the possibility of regulation by epigenetic modification.

## What Are the Molecular Reasons for Changes in the Telomere Motifs?

To explain telomere sequence change, the first candidate is the template subunit of telomerase, telomerase RNA (TR). The previously identified TR from yeast and vertebrates belongs to a different group of transcripts, whose connecting feature was that they were transcribed by RNA polymerase II (Pol II)—in all but ciliates; this used to be the single exception from Pol II transcripts before publication of the land plant TR identification [reviewed in (Podlevsky and Chen, 2016)]. By using the relatively long telomere motif of *Allium* to look for its TR within the total RNA sequence data pool, Fajkus et al. (2019) showed that a previously characterized noncoding RNA involved in the stress reaction in *A. thaliana,* called AtR8, was indeed the telomerase RNA subunit (Wu et al., 2012; Fajkus et al., 2019). It was a transcript of RNA polymerase III (Pol III) containing the corresponding regulatory elements in its promoter structure. For a long time, researchers expected that plant TR would be so divergent that it would be impossible to identify it based on a homology search (Cifuentes-Rojas et al., 2011). However, a certain degree of similarity was successfully used to identify a common TR in several *Allium* species with comparative Blast. Surprisingly, sequence homology, the presence of the same regulatory elements, and a corresponding template region led to the identification of TRs in *Allium*, *Arabidopsis* and more than 70 other distantly related plants, including those with diverged telomere motifs like *Genlisea*, *Cestrum*, and *Tulbaghia*. As far as we know, there is still no data on any algal TR, which would elucidate whether Pol III transcription of TR is a general feature for all plants or not. This work (Fajkus et al., 2019), based on CRISPR knock-out and other experiments, also showed that a previously identified telomerase RNA candidate in *A. thaliana* (Cifuentes-Rojas et al., 2011; Beilstein et al., 2012) was not a functional template subunit of telomerase, as was also demonstrated shortly after by (Dew-Budd et al., 2019). Assuming that the Pol II/Pol III dependency for TR transcription is a reliable evolutionary marker, future TR research in other main eukaryotic lineages will probably open new insights into the origin of eukaryotes. Telomerase genes and telomere sequences are unrecognized sources of information in this direction, and the finding of a Pol III dependent TR biogenesis pathway in ciliate and plant lineages may represent the first steps in this direction (Greider and Blackburn, 1989; Fajkus et al., 2019).

## How Did Chromosomes Become Linear?

A vast majority of prokaryotes contain circular chromosomes while linear chromosomes are the rule in eukaryotes. Therefore there are two possible scenarios in which either (i) linearization was performed by a primitive telomerase, preceding other processes which led to current linear chromosomal features and functions or (ii) linearization of a pre-eukaryotic circular chromosome was initially telomerase independent, but just before current eukaryotes diverged, a primitive telomerase started to occupy chromosome ends and became essential for the newly formed linear chromosomes (Nosek et al., 2006). Villasante et al. (2007) proposed an evolutionary scenario in which the breakage of the ancestral prokaryotic circular chromosome activated a transposition mechanism at DNA ends, allowing the formation of telomeres by a recombination-dependent replication mechanism: consequences of this hypothesis led to the surprising conclusion that eukaryotic centromeres were derived from telomeres.

Interestingly, the opposite process to linearization, i.e., formation of circular chromosomes (also termed ring chromosomes) has emerged from time to time during the evolution of eukaryotes, although being highly unstable. For example, in the case of *Amaranthus tuberculatus*, ring chromosomes appeared as a stress-induced response, carrying resistance against a herbicide (glyphosate); these extra ring chromosomes did not show hybridization with telomere probes in the karyotype analysis (Koo et al., 2018). The almost universal telomerase system and the exceptionality of circular chromosomes in eukaryotes do not allow us to support one hypothesis over the other. However, the recombinational machinery used in the alternative lengthening of telomeres (ALT), a telomerase-independent pathway, associated with certain human cancers (Zhang et al., 2019), is already present in prokaryotes. In addition, there is evidence of chromosome linearization occurring independently in distinct prokaryote lineages (Ferdows and Barbour, 1989; Nosek et al., 1995; Volff and Altenbuchner, 2000). Therefore, the hypothesis that the first linear eukaryotic chromosome (originating from a prokaryote ancestor) was telomerase-independent seems more likely. There are some examples that show that the telomerase-based system is not essential for telomere maintenance in all eukaryotes: retrotransposons in *Drosophila* telomeres, satellite repeats in *Chironomus*, another insect (Rubin, 1978; Biessmann and Mason, 2003), and ALT in telomerase-negative human cancers (Hu et al., 2016; Zhang et al., 2019). Yet, some of these systems may not be as different, and may perhaps share a common origin: in *Drosophila*, the telomere maintenance, based in retrotransposition, is not too distinct from the telomerase-based mechanism (Danilevskaya et al., 1998), leading to the hypothesis that the telomerase itself may be a former retrotransposon. But certainly, telomerase-negative plant species have not been discovered to date and all exceptions, in which the typical plant-type telomere was absent, were later shown to have different, but still telomerase-synthesized, motifs. Nevertheless, the ALT machinery is present in plants in parallel to the telomerase activity (Watson and Shippen, 2007; Ruckova et al., 2008). Interesting questions about the role of telomerase, telomeres and their maintenance in plant tumors arise from that. An attractive one is about the absence of metastasis in plants, despite the presence of ALT, perhaps related with plant tissue rigidity or different immune systems than in animals (Seyfried and Huysentruyt, 2013).

Although we are gaining increasing knowledge of telomere biology, we are still unable to explain the emergence of telomerase in eukaryotes. Current evidence supports the hypothesis that the emergence of eukaryotes together with their linear chromosomes, telomeres, and telomerase was related to the appearance of spliceosomal introns in archaeal hosts (Koonin, 2006; Fajkus et al., 2019). The similarity between TERT and other retroelements has been discussed for some time (Pardue et al., 1997). Remarkably, a relatively recent study showed that TERT, as a probable member of progeny group II introns, is sequentially close to *Penelope*-like element retrotransposons (Gladyshev and Arkhipova, 2007). But TERT is only one of the two essential telomerase components, and TR is, in its origin, even more enigmatic due to its low sequence conservation across all eukaryotes [see review (Podlevsky and Chen, 2016; Fajkus et al., 2019)].

## Conclusion

At the beginning of the plant genomics era, the telomere sequence was considered almost changeless. The general conservation of telomeres and the telomerase system suggested that all plants may have the TTTAGGG plant-type telomere. The identification of unusual telomere sequences in complex plant genomes, in many cases with giant C-values (such as in *Cestrum* and *Allium* sp.), was worth the effort, since the exceptionally long *Allium* telomere motif was the clue in looking for a genuine TR in land plants. The newly described TR in plants and further telomere/telomerase research in basal clades of algae might reveal valuable information about early evolution, therefore plant telomere research can significantly contribute to hypotheses on the emergence of eukaryotes.

## Author Contributions

VP and SG have contributed equally to the writing, editing, and preparation of this mini-review.

## Funding

This work was supported by ERDF [project SYMBIT, reg. no. CZ.02.1.01/0.0/0.0/15_003/0000477], EMBO Short-Term Fellowship 7368 to V.P., Spanish [CGL2016-75694-P (AEI/FEDER, UE)] and Catalan [grant number 2017SGR1116] governments. S.G. is the holder of a Ramón y Cajal contract (RYC-2014-16608).

## Conflict of Interest

The authors declare that the research was conducted in the absence of any commercial or financial relationships that could be construed as a potential conflict of interest.
